# U.S. Government engagement in support of global disease surveillance

**DOI:** 10.1186/1471-2458-10-S1-S13

**Published:** 2010-12-03

**Authors:** Rebecca L Katz, Leana M López, Joseph F Annelli, Ray R Arthur, Dennis Carroll, Leonard W Chapman, Kenneth Cole, Cyril G Gay, Daniel L Lowe, Gary Resnick, Kevin L Russell

**Affiliations:** 1 U.S. Department of State, Washington, DC, USA; 2 George Washington University, School of Public Health and Health Services, Washington, DC, USA; 3 U.S. Department of Agriculture, Riverdale, MD, USA; 4 U.S. Centers for Disease Control and Prevention, Atlanta, GA, USA; 5 U.S. Agency for International Development, Washington, DC, USA; 6 U.S. Department of Defense, Arlington, VA, USA; 7 U.S. Department of Agriculture, Beltsville, MD, USA; 8 U.S. Department of Energy, Los Alamos National Laboratory, Los Alamos, NM, USA; 9 U.S. Department of Defense, Silver Spring, MD, USA

## Abstract

Global cooperation is essential for coordinated planning and response to public health emergencies, as well as for building sufficient capacity around the world to detect, assess and respond to health events. The United States is committed to, and actively engaged in, supporting disease surveillance capacity building around the world. We recognize that there are many agencies involved in this effort, which can become confusing to partner countries and other public health entities. This paper aims to describe the agencies and offices working directly on global disease surveillance capacity building in order to clarify the United States Government interagency efforts in this space.

## Introduction

The United States Government is committed to supporting the establishment and sustainability of comprehensive global disease surveillance. Many agencies across the U.S. Government, working in a coordinated fashion in every region of the world, are engaged in this essential mission. This commitment is based on the recognition that a weakness in the surveillance system in one part of the world is a weakness for the entire globe, and that every nation needs the infrastructure to prepare for, respond to, and recover from health emergencies. This work is also in support of Article 44 of the Revised International Health Regulations [IHR (2005)], which directs countries that are able to provide assistance to other nations through technical cooperation and financial resources to develop, strengthen and maintain core disease surveillance capacities [1].

Several U.S. Government agencies have been engaged in efforts to support global disease surveillance for years, and in some cases, for decades. Agencies work together to coordinate efforts, ensure sustainable programs, and address the needs of partner countries. In this paper, we present an overview of each individual agency, their particular interests and strengths, and the focus of their operations. This overview is intended to help the international community better understand the U.S. Government partner agencies, how they operate, and how they differ from their partners.

## U.S. Government agencies engaged in supporting global disease surveillance

The following section provides a brief overview of each U.S. Government agency. Agencies are presented in alphabetical order.

### U.S. Agency for International Development

#### Emerging Pandemic Threats Program

The U.S. Agency for International Development (USAID) launched the Emerging Pandemic Threats (EPT) program [2] to aggressively preempt or combat diseases that could spark future pandemics by emphasizing early identification of, and response to, dangerous pathogens in animals before they can become significant threats to human health. Using a risk-based approach, the EPT program builds on USAID’s experience in disease surveillance, training, and outbreak response to focus on geographic areas where these threats are most likely to emerge. The EPT program draws on expertise from across the animal-and human-health sectors to build regional, national, and local capacities for early disease detection, laboratory-based disease diagnosis, rapid disease response and containment, and risk reduction. These efforts target a limited number of geographic areas, known as “hot spots,” where new disease threats have emerged in the past. The EPT program focuses on “hot spots” in the Congo Basin of East and Central Africa, the Mekong region and other “hot spots” in Southeast Asia, the Amazon region of South America, and the Gangetic Plain of South Asia.

USAID anticipates that the EPT program will develop predictive models for early identification of viral and other biological threats in “hot spot” regions and that it will enhance regional, national, and local capacities for surveillance, laboratory diagnosis, and field epidemiology in both the animal- and human-health sectors in these areas. The EPT program’s investments in disease detection and response will be reflected in their routine application in the management of more normative diseases in these areas, such as malaria, cholera, and meningitis. These contributions more broadly support the tenets outlined in the IHR(2005) and equivalent international health standards of the World Organization for Animal Health (OIE).

### U.S. Department of Agriculture

#### Agricultural Research Service

The Agricultural Research Service (ARS) [3] is the principal in-house research agency of the U.S. Department of Agriculture (USDA). ARS is actively engaged in implementing research programs that support global disease surveillance initiatives for plants and animals, including emerging diseases and zoonotic agents that pose a threat to human health. ARS research pro grams support disease surveillance initiatives in several U.S. Government agencies, such as USDA’s Animal and Plant Health Inspection Service (APHIS), the Centers for Disease Control and Prevention (CDC), and the State Department’s Biosecurity Engagement Program (BEP). In addition, ARS actively collaborates with international partners worldwide on research projects dedicated to support disease surveillance programs. ARS is one of the founding members of the Global Foot-and-Mouth Disease Research Alliance (GFRA), which has as its primary mission to support the United Nations’ Food and Agricultural Organization (FAO) and OIE global efforts to control and eradicate Foot-and-Mouth Disease (FMD).

#### Animal and Plant Health Inspection Service

USDA’s Animal and Plant Health Inspection Service (APHIS) [4] plays a significant role in increasing global food security by promoting technology- and science-based solutions and capacity-building activities in countries around the world. A cornerstone of this concept is enhanced coordination between the agriculture and public health sectors for disease surveillance, detection, and control. APHIS plays a direct role in public health, especially in efforts to mitigate veterinary diseases and ensure the healthfulness of agricultural practices and products. APHIS has programs in disease detection and surveillance, disease exclusion, animal disease information systems, and emergency response. Technical expertise is delivered through APHIS’s Centers for Epidemiology and Animal Health, the National Surveillance Unit, the National Veterinary Services Laboratory and the National Animal Health Laboratory Network.

### U.S. Department of Defense

#### Armed Forces Health Surveillance Center, Division of Global Emerging Infection Surveillance and Response

The Division of Global Emerging Infections Surveillance and Response (GEIS) of the Armed Forces Health Surveillance Center (AFHSC) [5] coordinates a global program of infectious disease surveillance under the five militarily relevant pillars of respiratory infections, gastrointestinal infections, antimicrobial resistant organisms, febrile and vector-borne infections, and sexually transmitted infections. In addition to surveillance activities, capacity building, training, and communication are important dimensions to the program. Currently, GEIS operates projects in over 80 countries through out the world, and Department of Defense overseas laboratories perform the majority of the work outside the U.S. from their fixed assets in Cairo, Egypt; Nairobi, Kenya; Bangkok, Thailand; Lima, Peru; and Southeast Asia.

GEIS releases requests for proposals, evaluated through a competitive proposal review process, with emphasis on identified gap areas. Recent emphases are on standardization, expanded military-to-military engagements, and fusion of surveillance activities into those of the host nations, thus enhancing capacity building efforts. Compliance with IHR(2005) provides the framework for these efforts, while fostering open, professional, and supportive relationships between U.S. Government and host country public health assets.

#### Chemical and Biological Defense Program

The Chemical and Biological Defense Program, [6] under the Assistant Secretary of Defense for Nuclear and Chemical and Biological Defense Programs, houses both the Joint Program Executive Office for Chemical Biological Defense and the Joint Science and Technology Office. The Joint Program Executive Office for Chemical Biological Defense supports the biosurveillance functional areas of global awareness, passive monitoring, active surveillance, pathogen identification, and pathogen characterization. The Joint Science and Technology Office works to develop detection systems, broad spectrum next generation diagnostics and disease models to support advancing surveillance systems. This office also focuses on enhancing early warning informatics, which includes data collection, reporting and analysis.

#### Defense Threat Reduction Agency, Cooperative Biological Engagement Program

The mission of the Defense Threat Reduction Agency’s (DTRA) Cooperative Biological Engagement Program (CBEP) [7] is to counter the threat posed by select agents, related materials, expertise, other emerging infectious disease risks, and to prevent these agents from reaching any state or non-state actors who may use them against the United States or its allies. The program focuses on delivering tailored approaches that recognize and build upon partner countries’ indigenous capacities. The CBEP mission is achieved through execution of three key product lines: Biological Safety & Security capacity building; Disease Surveillance, Detection, Diagnosis, and Reporting; and Cooperative Biological Research. Through these three product lines the Program aims to secure dangerous pathogens; promote open and active disease reporting and response; and advance transparent research to understanding pathogens and developing potential countermeasures. CBEP works with a number of partner countries to assist in compliance with the IHR(2005), the OIE reporting guidelines, and the FAO reporting guide lines. CBEP also works with partner countries to implement a disease surveillance, detection, diagnosis, reporting, and response system that is safe, secure, and sustainable; and to integrate existing surveillance efforts to improve global disease surveillance and ensure timely, accurate situational awareness of infectious disease threats.

### U.S. Department of Energy

#### Los Alamos National Laboratory

Los Alamos National Laboratory (LANL) [8] is a premier national security research institution under the Department of Energy (DOE), delivering scientific and engineering solutions for crucial and complex problems. A broad multi-agency research and development program is pursued at LANL in support of global infectious disease surveillance. These efforts include the development and application of enabling information science and technology, high throughput bioanalytical systems, characterization of host-pathogen interactions, and field deploy-able detection and identification systems.

The Department of Energy Joint Genome Institute, of which LANL is a principal component, is a major national microbial genome sequencing resource that has completed over 30 percent of the bacterial genomes sequenced nationwide. The High Throughput Laboratory Network (HTLN) is a joint effort with the University of California Los Angeles, and is designed to process and analyze hundreds of samples per day in order to support global surveillance of infectious diseases of both health and security concern and to establish a global network for information/data management, sharing and knowledge generation. High performance computing resources support immune and epidemiological modeling and pathogen feature recognition and pathogen signature detection assay design. Through its many collaborations, LANL strives to develop efficacious cost-effective surveillance technologies and an enabling knowledge base to achieve a sustainable global infectious disease surveillance network.

### U.S. Department of Health and Human Services

The Department of Health and Human Services (HHS) is the technical agency for human health, disease surveillance, and the public health response to emergencies and disasters. The IHR(2005) Program sits in The Office of the Assistant Secretary for Preparedness and Response (ASPR) within HHS, [9] and works closely with the official National Focal Point for the United States- the Operations Center for the Office of the Secretary of HHS. The Centers for Disease Control and Prevention (CDC) — programs described below—is part of HHS.

#### Centers for Disease Control and Prevention

##### Global Disease Detection Program

The Global Disease Detection program in the Division of Global Disease Detection and Emergency Response [10] is CDC’s principal program for developing and strengthening global public health capacity to rapidly identify and contain naturally occurring or man-made disease threats from around the world. The program presently comprises eight international regional centers and CDC headquarters components. The GDD regional centers, located in every WHO region of the world, work with country partners to implement disease detection and response trainings, protocols, and interventions. The centers’ expertise includes training in field epidemiology and laboratory methods, surveillance and response for emerging infectious disease threats, assistance with pandemic influenza preparedness, promotion of zoonotic disease investigations and control efforts, risk communications, and laboratory biosafety and improved laboratory systems. A cadre of US-based CDC scientists provides technical expertise to the regional centers. In recognition of GDD’s capacity development activities, WHO designated GDD as a Collaborating Center for Implementation of IHR National Surveillance and Response Capacity in December 2009.

An integral part of GDD is the GDD Operations Center located at CDC headquarters. This innovative epidemic intelligence and response operations unit uses non-traditional surveillance methods and information from internationally-based CDC staff and partners to provide early warning about international disease threats thereby positioning CDC to respond rapidly when assistance is requested. CDC is a key partner in Global Outbreak Alert Response Network (GOARN) and participation of CDC staff in GOARN teams is coordinated by the GDD Operations Center.

##### Division of Public Health Systems and Workforce Development

Like the Global Disease Detection and Emergency Response Division, the Division of Public Health Systems and Workforce Development [11] sits in the Center for Global Health at CDC. The purpose of this division is to work with Ministries of Health and other public health partners to build strong public health systems and workforce capacity. The Division operates several training programs, including the Field Epidemiology Training Program (FETP), the Field Epidemiology and Laboratory Training Program (FELTP), and the Sustainable Management Development Program. FETPs and FELTPs are typically Ministry of Health-based in-service training programs in applied epidemiology aimed at strengthening countries’ epidemiology, surveillance, outbreak response, and laboratory systems and workforce. As of mid-2010, the division is supporting 15 FETP/FELTPs covering 29 countries with another 8 programs covering 11 countries currently in development. An additional 20 programs, previously supported by CDC, are operating independently.

The division is also collaborating extensively with the World Health Organization’s Regional Office for Africa (WHO/AFRO) on implementing the Integrated Disease Surveillance and Response (IDSR) strategy. The IDSR strategy aims to improve surveillance and response capacity in African countries and is WHO/AFRO’s primary strategy for improving early detection and response to infectious disease outbreaks.

### U.S. Department of State

#### Biosecurity Engagement Program

The U.S. Department of State manages America’s relationships with foreign governments, international organizations, and the people of other countries. The Biosecurity Engagement Program (BEP) [12] seeks to engage biological scientists and combat biological threats worldwide by providing assistance to improve biosecurity and biosafety, conduct cooperative research, and improve infectious disease detection and surveillance. Specifically, BEP provides assistance in: biosafety and biosecurity, through technical consultations, risk assessments, and training courses that build human capacity and internal expertise to create a sustainable culture of laboratory biorisk management; disease detection and control, by strengthening the capacity for public health and veterinary health systems to detect, report, and control infectious disease outbreaks; scientist engagement, by encouraging a safe, secure and sustainable bioscience capacity through joint scientific collaborations designed to help prevent, detect, and respond to biological threats; and sustainable capacity, with a focus on long-term sustainability and capacity building that creates an infrastructure for biorisk management.

BEP leverages technical resources and experts from numerous U.S. agencies, universities, international organizations, NGOs, and the National Academies of Sciences to meet its core objectives. In addition to inter-agency and organizational collaborations, BEP works closely with host-country governments, U.S. Embassies, and other nations to identify needs and implement assistance necessary to ensure safe, secure, and sustainable bioscience capacity, while achieving the larger goal of reducing global biological risks. BEP efforts are threat-driven and designed to prevent, detect, and respond to both existing and emerging global biological threats.

## Discussion

Effective global engagement in support of disease surveillance capacity building requires coordination and partnership. Each of the above-mentioned agencies and offices has unique resources and technical expertise. These entities first must coordinate with each other and then with international partners to ensure that global engagement efforts are efficient, build off of each other’s expertise, and are best combined to promote effective disease surveillance capacity building. This internal co-ordination is not without challenges, as each entity has different organizational cultures, administrative processes, and substantive expertise. Agencies do, however, actively try to work around these challenges, and use the White House National Security Staff as an overarching coordinating body.

In addition to internal U.S. coordination, agencies require a strong working relationship with partner countries in order to be most effective. Ideal country engagement involves a commitment on the part of the partner country to an agreed upon strategy for engagement that includes planning for financial sustain ability and human resources needs. Effective engagement also requires the designation of points of contact for coordinating efforts, and a commitment to open communication with the assisting entity. Additionally, it requires that both the U.S. and its partner country recognize that their individual perception of needs and priorities may differ, and that strong relationships and communications are essential to successful engagement.

## Conclusion

The U.S. is committed to providing resources and expertise to enhance global disease surveillance and assist in building a comprehensive, effective and efficient public health infrastructure. Each U.S. Government agency that is engaged in building global disease surveillance capacity brings its own particular expertise and interest to the endeavor. These agencies, however, work closely in coordination both with other U.S. entities as well as with partner countries and organizations, building on individual expertise.

## Abbreviations

AFHSC: Armed Forces Health Surveillance Center; APHIS: Animal and Plant Health Inspection Service; ARS: Agricultural Research Service; ASPR: Assistant Secretary for Preparedness and Response; BEP: Biosecurity Engagement Program; CBEP: Cooperative Biological Engagement Program; CDC: Centers for Disease Control and Prevention; DOE: Department of Energy; DTRA: Defense Threat Reduction Agency; EPT: Emerging Pandemic Threats Program; FAO: Food and Agricultural Organization; FELTP: Field Epidemiology and Laboratory Training Program; FETP: Field Epidemiology Training Program; FMD: Foot-and-Mouth Disease; GDD: Global Disease Detection Program; GEIS: Global Emerging Infections Surveillance and Response; GFRA: Global Foot-and-Mouth Disease Research Alliance; GOARN: Global Outbreak Alert Response Network; HHS: Department of Health and Human Services; HTLN: High Throughput Laboratory Network; IHR: International Health Regulations; LANL: Los Alamos National Laboratory; OIE: World Organization for Animal Health; USAID: United States Agency for International Development; USDA: United States Department of Agriculture.

## Competing interests

No competing interests to declare.

## Authors’ contributions

All authors contributed equally to the text.
